# A Case Series of Serious Adverse Events Following Anti-VEGF Intravitreal Injections

**DOI:** 10.22336/rjo.2025.70

**Published:** 2025

**Authors:** Andreea Alexandra Mihaela Muşat, Nicoleta Zamfiroiu-Avidis, Cornel Ştefan, Schmitzer Andreea, Gabriela Muşat, Gabriela Udrea, Ioana Stella Popescu, Ovidiu Muşat

**Affiliations:** 1 “Carol Davila” University of Medicine and Pharmacy, Bucharest, Romania; 2 Clinical Emergency Eye Hospital, Bucharest, Romania; 3 “Dr. Carol Davila” Central Military Emergency University Hospital, Bucharest, Romania; 4 Apel Laser SRL, Ilfov, Romania

**Keywords:** anti-VEGF, endophthalmitis, cataract, retinal detachment, cataract, anti-VEGF = anti-vascular endothelial growth factor, AMD = age-related macular degeneration, BCVA = best corrected visual acuity, OD = right eye, OS = left eye, IOP = intraocular pressure, IOL = intraocular lens, RRD = rhegmatogenous retinal detachment

## Abstract

**Purpose:**

To highlight serious adverse effects regarding intravitreal anti-vascular endothelial growth factor (anti-VEGF) therapy, which is widely used for the treatment of retinal diseases, including two cases of post-injection endophthalmitis, one of which was complicated by rhegmatogenous retinal detachment, and one case of secondary cataract following a potentially unnecessary injection.

**Methods:**

A retrospective analysis of three cases that developed complications after intravitreal anti-VEGF therapy.

**Results:**

All cases resulted in a decline in best corrected visual acuity (BCVA) that required additional surgical procedures.

**Discussion:**

While intravitreal anti-VEGF therapy has become the standard in the treatment of various retinal pathologies, it is not without risks. This case series presents significant adverse outcomes, emphasizing the potential for severe anatomical and functional consequences. As the global volume of anti-VEGF intravitreal injections increases, so must our commitment to patient safety, precision in diagnosis, and ethical decision making.

**Conclusion:**

Although generally safe and commonly used in clinical practice, physicians must be aware of the risks of anti-VEGF therapy and must remain vigilant regarding patient selection and risk-benefit considerations.

## Introduction

Intravitreal anti-VEGF therapy is routinely used for various retinal conditions, including wet age-related macular degeneration (AMD) [1,2], retinal vein occlusion, myopic choroidal neovascularization, and diabetic retinopathy [3]. Even though the therapeutic efficacy of various anti-VEGF agents has been thoroughly studied and proven, the adverse effects exist and may have devastating consequences regarding BCVA [4]. The most common adverse events include subconjunctival hemorrhage, vitreous hemorrhage, increased intraocular pressure, endophthalmitis, rhegmatogenous retinal detachment, uveitis, and traumatic cataract [2]. Even though most of them are minor and do not require further medical intervention [5], serious, although rare, complications such as endophthalmitis, retinal detachment, and cataract often require a surgical approach. The case series aims to highlight serious complications of anti-VEGF therapy with a focus on the required surgical intervention for resolution.

## Case Descriptions

### Case one: Endophthalmitis following intravitreal anti-VEGF therapy for wet AMD

**Fig. 1:**
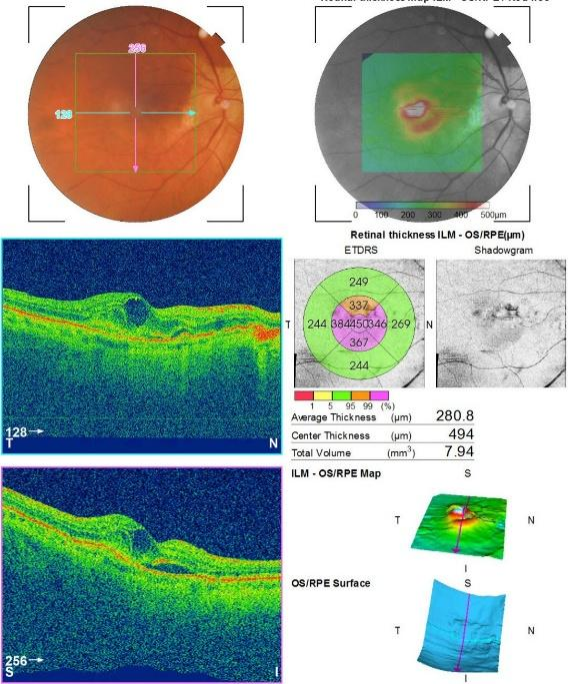
Macular OCT in OD. Macular edema, neurosensory retinal detachment, disorganization of retinal layers, and disruption of the normal vascular choroidal architecture are observed

**Fig. 2:**
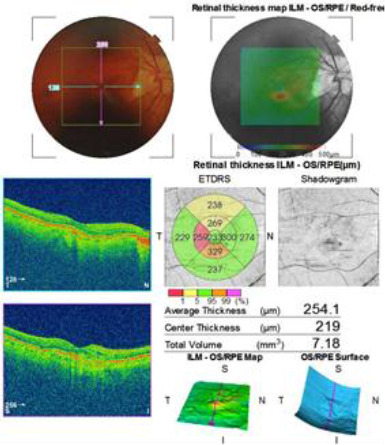
Macular OCT of the right eye (OD). The image shows the absence of intraretinal fluid and an improvement in local tissue architecture

Patient S.E., male, age 77 years old, had a history of previous intravitreal injections with anti-VEGF therapy (Aflibercept) in the right eye (OD) for the treatment of the macular edema associated with wet-AMD, the last one being administered 3 days before presentation. **Fig. 1** shows the macular OCT in OD obtained before the last intravitreal injection. He presented to the emergency department, complaining of decreased visual acuity and eye pain in OD. The ophthalmological examination revealed mixed conjunctival hyperemia, corneal edema, cells, and flare in the anterior chamber and pseudophakia in the posterior chamber. Fundus examination was not possible due to inflammatory exudates. B-scan ultrasonography showed inflammatory vitreous echoes and an attached retina. BVCA was hand motion with an intraocular pressure (IOP) within normal limits. Management included emergency vitrectomy, and a pathological sample from the vitreous body and anterior chamber was collected for microbiological culture, which revealed the presence of Staphylococcus epidermidis. The patient underwent endotamponade with air. Broad-spectrum antibiotics were administered intravitreally and systemically. Topical and systemic steroid and non-steroidal anti-inflammatory therapy were administered. One month postoperatively, BVCA in OD was 0.5. As the wet-AMD continued to advance, the patient began to complain about a decline in visual acuity. Further injections with anti-VEGF were administered with no further infectious complications. **Fig. 2** presents the macular OCT after the last intravitreal injection.

### Case two: Endophthalmitis and rhegmatogenous retinal detachment following intravitreal anti-VEGF therapy

Patient C.E., age 65 years old, presented to the emergency department for ocular pain and a rapid decline in visual acuity in the left eye (OS). She had previously received intravitreal therapy with Aflibercept and Triamcinolone for cystoid macular edema, the last injection being administered three days before presentation. The visual acuity in OS was light perception with intraocular pressure within normal limits (20 mmHg). Ophthalmological examination revealed mixed conjunctival hyperemia, corneal edema, cells, flare, and hypopyon in the anterior and posterior chamber pseudophakia. Fundus examination was not possible. B-scan ultrasonography demonstrated inflammatory vitreous signals with an attached retina. An emergency pars plana vitrectomy was performed, with vitreous and anterior chamber samples collected for microbiological analysis. Intraoperatively, a rhegmatogenous retinal detachment was observed, and silicone oil endotamponade was performed during surgery. Management included intravitreal and systemic administration of broad-spectrum antibiotics, as well as topical and systemic corticosteroid therapy. One month postoperatively, the BCVA in OS was 0,1 with normal IOP and no signs of inflammation at examination. Silicone oil removal was planned at a later date.

### Case three: Secondary cataract from anti-VEGF therapy for macular edema

**Fig. 3:**
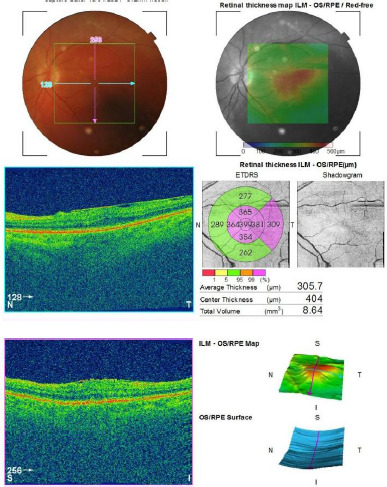
Macular OCT of the left eye (OS) showing a tractional epiretinal membrane associated with macular edema

Patient A.E., female, age 66 years, presented for an acute and progressive worsening of vision in OS, the onset of symptoms occurring six days before presentation. She was previously diagnosed with open-angle glaucoma in both eyes (AO) for which she was receiving therapy with latanoprost and had a history of 10 intravitreal injections with anti-VEGF in OS for macular edema, the last one being administered 10 days before presentation. Visual acuity in OS was hand motion, and an IOP of 14 mmHg. Ophthalmological examination revealed an advanced secondary cataract in the OS due to intravitreal therapy. Fundus examination was not possible due to the advanced cataract, and B-scan echography was performed, showing an attached retina. It was decided to proceed with surgical intervention, performing phacoemulsification, and a monofocal intraocular lens (IOL) was implanted in the capsular bag. One week post-intervention, further investigations were conducted. Visual acuity in OS was 0,4 with normal IOP on latanoprost. Fundus examination and Macular Optical Coherence Tomography showed epiretinal membrane with tractional macular edema (as shown in **Fig. 3**) in the OS, a condition for which intravitreal anti-VEGF therapy is not indicated. The patient was subsequently scheduled for surgical intervention for epiretinal membrane removal.

## Discussion

Although rare, with an incidence between 0.01 and 0,26%, endophthalmitis remains one of the most serious complications after anti-VEGF therapy [6]. The procedure should be performed under strict aseptic conditions, ideally in a very controlled environment, such as the operating room [7]. To minimize the risk of contamination, all personnel involved and the patient should wear face masks [8]. The patient’s ocular periocular area should be thoroughly disinfected, and a sterile drape should be applied to isolate the operative field. Sterile instruments, gloves, and single-use needles and syringes must be used.

While routinely used in clinical practice, recent meta-analyses show that topical antibiotic prophylaxis has not been proven to reduce the risk and might even be associated with an increased risk of postoperative infection [9,10]. Conversely, the use of povidone-iodine remains a critical step in preventing exogenous endophthalmitis in the context of intravitreal injections, with its efficacy well supported by the literature [11].

While it is known that cataract progression is a well-documented side effect with intravitreal steroids, it is unclear whether anti-VEGF therapy has a role in the formation or progression of lens opacities [12]. Secondary cataract in the context of anti-VEGF therapy mainly results from the puncture of the lens, an infrequent complication, with a reported incidence of 0.006%. Hyperopic eyes were more likely to be affected [13]. A traumatic cataract can rapidly develop after an object penetrates the lens [14]. Although the optimal intravitreal injection technique remains a subject of debate, current recommendations advise that the site of the injection should be located 3.5 mm from the limbus in aphakic or pseudophakic patients and 4 mm from the limbus in phakic patients, allowing entry through pars plana and sufficient distance to avoid inadvertent injury of the crystalline lens [13,15]. The needle, no larger than 27 gauge, should be pointing to the center of the eyeball. The “tunneled” technique involves introducing the needle into the sclera at an initial angle of approximately 30 degrees, followed by repositioning it perpendicularly toward the center of the globe. This approach enhances wound sealing upon needle withdrawal and reduces the risk of vitreous reflux or microbial entry [15,16].

Retinal tears and rhegmatogenous retinal detachments (RRDs) are other rare but severe complications of intravitreal injections. The injection site (3,5 or 4 mm from the limbus, depending on the lens status) should be precise to avoid the vitreous base. A more posterior or steep approach risks retinal injury, whereas an oblique entry reduces vitreous incarceration. Underlying retinal conditions and previous surgeries may predispose to the formation of retinal tears and RRDs [17]. There seems to be no association between RRD risk and physician experience, injection site, the substance used, or needle caliber [17,18]. RRDs are typically associated with poor visual outcomes at one-year follow-up [19].

Furthermore, considering the widespread use of intravitreal injections worldwide, understanding the reasons behind inappropriate administration, particularly in patients without indication, is essential in reducing such occurrences. These mistakes may erode the patient’s confidence and damage the therapeutic alliance between the physician and the patient. A standardized, team-based approach that incorporates rigorous safety protocols will likely be needed to reduce such events [20]. Managing macular edema effectively requires recognizing the underlying mechanism. When traction is involved, for example, in the case of a tractional epiretinal membrane and vitreomacular traction syndrome, vitreoretinal surgery is the treatment of choice. At the same time, anti-VEGF therapy alone is not sufficient [21,22]. Proper knowledge of the indications for intravitreal anti-VEGF therapy is essential for all clinicians performing these procedures. Administering such treatment without clear justification exposes the patient to unnecessary risks, including adverse events that may lead to permanent vision loss. Moreover, overtreatment not only poses physical dangers but also imposes psychological and financial burdens on the patient. Restricting intravitreal injections to well-established indications, confirmed through thorough clinical evaluation and appropriate imaging, is a fundamental aspect of responsible and ethical medical practice.

## Conclusion

This case series highlights serious but relatively uncommon complications associated with intravitreal therapy, including two cases of endophthalmitis, one complicated by rhegmatogenous retinal detachment, and one case of secondary cataract following an injection given for a questionable indication. While anti-VEGF therapy has revolutionized the management of multiple retinal diseases, these cases underscore the need for strict adherence to sterile technique, accurate patient selection based on established clinical indications, and careful post-injection monitoring. Awareness of potential complications and the implementation of preventive strategies are crucial to optimizing patient care and achieving the best possible visual outcomes in clinical practice.
